# Anemonin reduces hydrogen peroxide-induced oxidative stress, inflammation and extracellular matrix degradation in nucleus pulposus cells by regulating NOX4/NF-*κ*B signaling pathway

**DOI:** 10.1186/s13018-023-03679-8

**Published:** 2023-03-11

**Authors:** Zhijia Ma, Pengfei Yu, Xiaochun Li, Feng Dai, Hong Jiang, Jintao Liu

**Affiliations:** Department of Orthopaedic Surgery, Suzhou TCM Hospital Affiliated to Nanjing University of Chinese Medicine, No. 889, Wuzhong West Road, Gusu District, Suzhou, 215009 Jiangsu China

**Keywords:** Anemonin, Oxidative stress, Inflammation, Extracellular matrix, Nucleus pulposus cells

## Abstract

**Background:**

Excessive oxidative stress plays a critical role in the progression of various diseases, including intervertebral disk degeneration (IVDD). Recent studies have found that anemonin (ANE) possesses antioxidant and anti-inflammatory effects. However, the role of ANE in IVDD is still unclear. Therefore, this study investigated the effect and mechanism of ANE on H_2_O_2_ induced degeneration of nucleus pulposus cells (NPCs).

**Methods:**

NPCs were pretreated with ANE, and then treated with H_2_O_2_. NOX4 was upregulated by transfection of pcDNA-NOX4 into NPCs. Cytotoxicity was detected by MTT, oxidative stress-related indicators and inflammatory factors were measured by ELISA, mRNA expression was assessed by RT-PCR, and protein expression was tested by western blot.

**Results:**

ANE attenuated H_2_O_2_-induced inhibition of NPCs activity. H_2_O_2_ enhanced oxidative stress, namely, increased ROS and MDA levels and decreased SOD level. However, these were suppressed and pretreated by ANE. ANE treatment repressed the expression of inflammatory factors (IL-6, IL-1*β* and TNF-*α*) in H_2_O_2_-induced NPCs. ANE treatment also prevented the degradation of extracellular matrix induced by H_2_O_2_, showing the downregulation of MMP-3, 13 and ADAMTS-4, 5 and the upregulation of collagen II. NOX4 is a key factor regulating oxidative stress. Our study confirmed that ANE could restrain NOX4 and p-NF-*κ*B. In addition, overexpression of NOX4 counteracted the antioxidant and anti-inflammatory activities of ANE in H_2_O_2_-induced NPCs, and the inhibition of the degradation of extracellular matrix induced by ANE was also reversed by overexpression of NOX4.

**Conclusion:**

ANE repressed oxidative stress, inflammation and extracellular matrix degradation in H_2_O_2_-induced NPCs by inhibiting NOX4/NF-*κ*B pathway. Our study indicated that ANE might be a candidate drug for the treatment of IVDD.

## Introduction

Low back pain (LBP) is a common clinical disease, followed by a huge family health burden and social and economic burden [1]. About 70%-80% of people have to experience LBP at least once in their life [2]. Intervertebral disk degeneration (IVDD) is considered to be one of the prime reasons of LBP [3]. The intervertebral disk structure includes the endplates, the peripheral concentric fibrosis and the nucleus pulposus [4]. The nucleus pulposus (NP) tissue is the main functional structure of the intervertebral disk, whose tolerance to harmful stimuli is less than that of the fibrous ring and endplate [5]. NP degeneration plays a more and more important role in IVDD [6, 7]. Degeneration of NP is manifested by the loss of extracellular matrix and collagen, proteoglycan and the upregulation of matrix metalloproteinases (MMPs) family [8]. However, the pathological mechanism of NP degeneration is complex and has not been clearly defined. Hence, the study of the pathological mechanism of NP degeneration is critical for the potential therapeutic strategies of IVDD.

Oxidative stress refers to the downregulation of antioxidant defense system function in the body, thus Large amount of ROS is accumulated [8], which can amplify apoptosis and aging of nucleus pulposus cells (NPCs) and enhance inflammation, and then eventually cause to IVDD [9]. Moreover, the apoptosis rate was obviously reduced by downregulating the expression of ROS in human NPCs [10]. The NOX family consists of seven members (NOX1-5 and DUOX1-2), of which NOX4 possesses the wide distribution of cell types [11]. It has been reported that NOX4 existed in NPCs and participated in modulating cell senescence [12]. Knockdown of NOX4 gene could significantly slow down the IVDD [13].

Multiple proinflammatory factors (e.g., IL-6, IL-1*β* and TNF-*α*) were significantly increased in the development of IVDD [14]. These proinflammatory factors could further induce the production of MMPs and ADAMSTs, resulting in excessive decomposition of extracellular matrix and accelerating IVDD [14]. NF-*κ*B widely distributed in cytoplasm and plays a crucial role in regulating oxidative stress and inflammatory [15]. NOX4 could activate NF-*κ*B pathway, and then upregulated the inflammatory factors and oxidative stress [16, 17]. Therefore, reducing inflammation and oxidative stress in NPCs by reducing the NOX4/NF-*κ*B axis is a key strategy for treatment of IVDD.

Anemonin (ANE) mainly exists in *Ranunculaceae* and *Gramineae plants* (such as *Ranunculus*, *Pulsatilla*, *clematis*, *citronella root* and *Anemone japonica*) [18]. ANE has multiple biological activities, such as anti-bacterial, anti-inflammatory and antioxidation [19–21]. ANE extracted from *Clematis* could significantly alleviate the inflammation of rheumatoid arthritis through percutaneous administration [20]. ANE showed neuroprotective effect by the antioxidant activity and inhibiting apoptosis [22]. Nevertheless, the effect of ANE in IVDD is still unclear.

In our study, we explored the effect and related mechanism of ANE on IVDD in vitro. NPCs degeneration was induced by H_2_O_2_ [23–25], and then, the effects of ANE on oxidative stress, extracellular matrix degeneration and inflammation were assessed. Mechanistically, the effect of NOX4/NF-*κ*B on the protective effect of ANE on NPCs was also explored.

## Materials and methods

### Cell culture

NP tissues were obtained from 12 patients (gender, 5 women and 7 men; Pfirrmann grade, 3 III, 5 IV and 4 V; average age, 37 years; range 21–63 years) with IVDD at Suzhou TCM Hospital Affiliated to Nanjing University of Chinese Medicine for surgical treatment. All patients were informed and signed the informed consent form. Our research was approved by ethics committee of Suzhou TCM Hospital Affiliated to Nanjing University of Chinese Medicine (No: S377). Primary NPCs were isolated and cultured according to previous study [25]. Primary NPCs were cultured in DMEM/F12 medium including 10% fetal bovine serum, 100 U/ml penicillin and 100 mg/L streptomycin. The cell culture condition was at 37 °C, 5% CO_2_ in an incubator. The NPCs in second generation were used for subsequent experimental studies. Nucleus pulposus cells were verified by performing immunofluorescence staining of aggrecan and collagen II.

### Cell treatment

NPCs were inoculated into 96-well plates at the concentration of 5 × 10^3^/well. After 24 h, the cells were treated with H_2_O_2_ (0, 25, 50, 100, 200, 500, 1000 μM) for 24 h or ANE (0, 1, 2, 5, 10, 20, 50, 100 μM) for 48 h. Cytotoxicity was detected by MTT method.

After 24 h in 96-well plates. NPCs were pretreated with ANE (0, 2, 5, 10 μM) [26, 27] for 24 h, and then treated with H_2_O_2_ (200 μM) for another 24 h. pcNDA-NOX4 was transfected into NPCs using Lipofectamine 2000 according to the application manual, which was applied to upregulate the expression of NOX4.

### MTT assay

NPCs (5 × 10^3^/well) were inoculated into 96-well plates and cultured overnight. The cells were treated according to the above method. Then, 50 μl MTT reagent was added to each well, and the cells were incubated for 4 h at 37 °C. After discarding the supernatant, 150 μl/well of dimethyl sulfoxide (DMSO) solution was added into each well, and then the plates were shocked for three times (30 s each time). The absorbance value of 570 nm was detected with a microplate reader.

### ROS assay

The ROS level in NPCs was measured through the ROS test kit (Beijing Bioteke Company) according to the introduction. After washed twice with sterile PBS, the cells were treated with 10 μM DCFH-DA for 20 min at 37 °C in dark, and turned every 4 min. Then, the fluorescence intensity of DCFH-DA was assessed.

### ELISA assay

NPCs (1 × 10^5^/well) were inoculated into 6-well plates and cultured overnight. Then, the cells were treated for 24 h according to the previous description. Then, malondialdehyde (MDA), superoxidedismutase (SOD), interleukin-6 (IL-6), interleukin-1*β* (IL-1*β*) and tumor necrosis factor-*α* (TNF-*α*) contents were measured according to the instructions of the corresponding kit.

### RT-PCR

Total RNA of NPCs was isolated with Trizol lysate. The extracted RNA was reverse-transcribed into cDNA according to the instructions of cDNA synthesis kit. Synthetic cDNA, as template, was mixed with 0.4 μl of ROX Reference Dye II, specific upstream and downstream amplification primers (0.8 μl) and 10 μl of SYBR® Premix EX TaqTM II to construct RT-PCR system. RT-PCR was performed according to the following procedure: 95 °C for 30 s, cycle once; 95 °C for 5 s, 60 °C for 30 s, 40 cycles. *β*-actin was taken as the internal parameter, and the mRNA level of the target gene was evaluated by 2^−ΔΔct^. The primer sequence was as follows: *β*-actin, F: 5′-CACCATTGGCAATGAGCGGTTC-3′, R: 5′-AGGTCTTTGCGGATGTCCACGT-3′); NOX4, F: 5′-TGTTGG‐GCCTAGGATTGTGTT-3′ and R: 5′-AGGGACCTTCTGTGATCCTCG-3′; MMP-3, F: 5′-ATGATGAACGATGGACAGATGA-3′ and R: 5′-CATTGGCTGAGTGAAAGAGACC-3′; MMP-13, F: 5′-GGCCAGAACTTCCCAACCA-3′ and R: 5′-ACCCTCCATAATGTCATACCC-3′; ADAMTS-4, F: 5′-ACCCAAGCATCCGCAATC-3′ and R: 5′-CAGGTCCTGACGGGTAAACA-3′; ADAMTS-5, F: 5′-CGACAAGAGTCTGGAGGTGAG-3′ and R: 5′-CGTGAGCCACAGTGAAAGC-3′; collagen II, F: 5′-GGCAATAGCAGGTTCACGTACA-3′ and R: 5′-CGATAACAGTCTTGCCCCACTT-3′;

### Western blot

NPCs were seed in 6-well plates. When cell number reached 5 × 10^6^/well, 200 μL RIPA reagent/well was used to extract the total proteins of NPCs in different treatment groups, and the proteins were quantitatively analyzed with BCA protein detection kit according to the instruction. The same amount of protein sample was separated through 12% SDS-PAGE, and then, the protein was electrically transferred to PVDF membranes. The membranes were blocked with 5% skimmed milk powder, and then, the corresponding primary antibody was added for overnight incubation at 4 °C. The membranes were added with secondary antibody and incubated for 1.5 h. ECL solution was added for visualizing the bands, GAPDH was used as internal reference, and Image J software was used for quantitative analysis of protein gray. The primary antibodies used in the experiment included: MMP-3 (ab52915, Abcam, 1:1000), MMP-13 (ab51072, Abcam, 1:1000), ADAMTS-4 (ab84792, Abcam, 1:1000), ADAMTS-5 (ab41037, Abcam, 1:1000), collagen II (ab188570, Abcam, 1:1000), NOX4 (ab154244, Abcam, 1:1000), NF-*κ*B (ab32536, Abcam, 1:1000), p-NF-*κ*B (ab76302, Abcam, 1:1000), GAPDH (ab181602, Abcam, 1:1000). The second antibody used in the experiment included: Goat Anti-Rabbit IgG H&L (1:5000, ab96899, Abcam, Goat Anti-Mouse IgG H&L (1:5000, ab96879, Abcam).

### Statistical analyses

The data was analyzed by SPSS 22.0 software. The results are expressed in mean ± standard deviation (SD). One-way ANOVA followed by Tukey’s test is used for three or more groups comparison. *P* < 0.05 was statistically significant. Every experiment was performed at least three independent measurements.

## Results

### ANE resisted H_2_O_2_-induced inhibition of NPCs activity

**Fig. 1:**
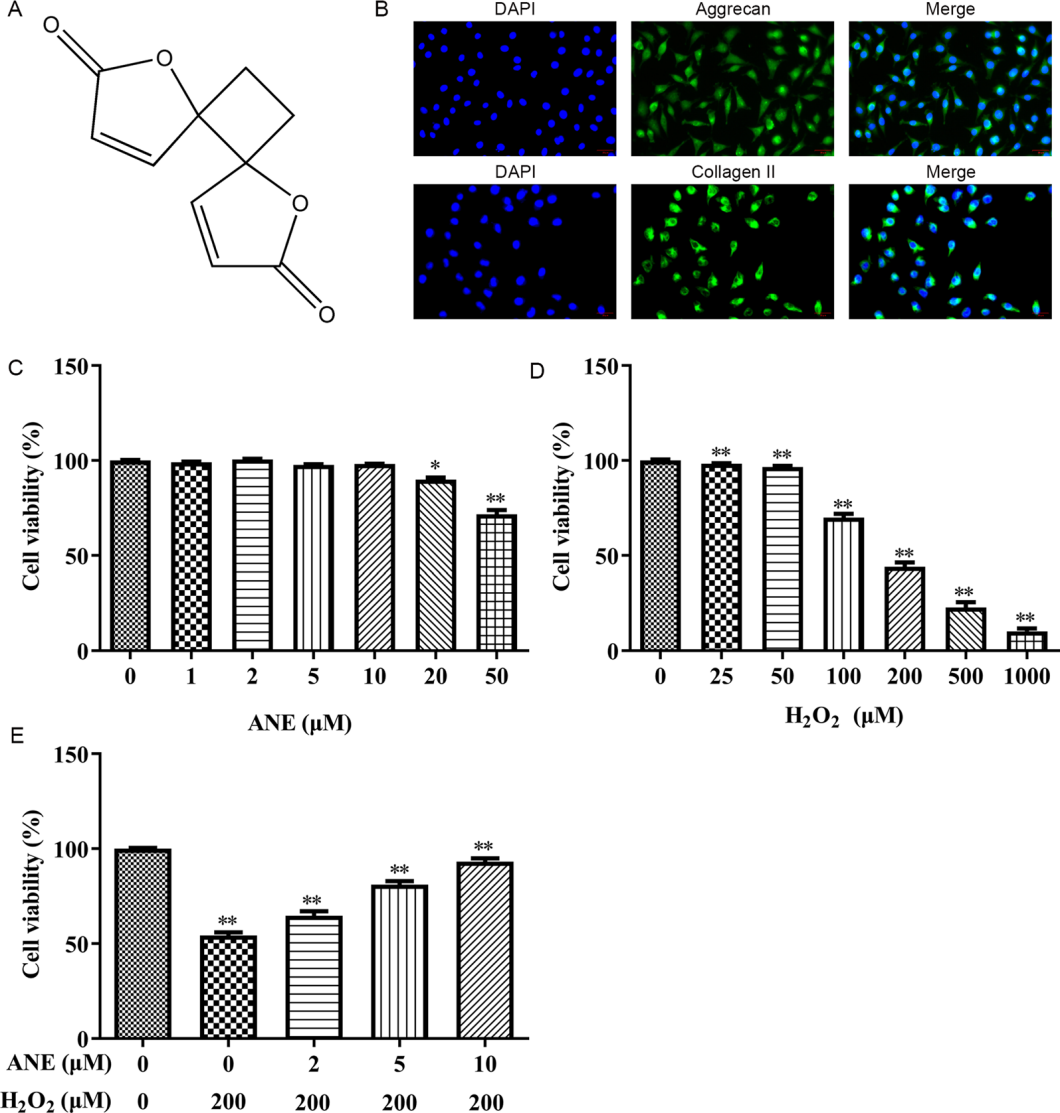
ANE repressed the cytotoxic induced by H_2_O_2_ in NPCs. **A** The chemical formula of ANE is shown. **B** Immunofluorescence detection of aggrecan and collagen II was performed in isolated cells from IVDD tissues. **C** After treatment with ANE for 48 h, cell viability was measured by MTT assay. **D** After treatment with H_2_O_2_ for 24 h, cell viability was detected by MTT assay. **E** After treatment with or without ANE for 24 h, the cells were treated with or without H_2_O_2_ for 24 h, and then the cell viability was assessed. *p < 0.05 versus control group, **p < 0.01 versus control group

The chemical formula of ANE is exhibited in Fig. 1A. Our finding indicated that aggrecan and collagen II were expressed in more than 95% of cells, which confirmed the cells obtained were NPCs (Fig. 1B). Subsequently, we explored the cytotoxicity of ANE and its protective effect in H_2_O_2_-induced NPCs. ANE (0, 1, 2, 5, 10, 20, 50 μM) was used to treat NPCs for 48 h. The results showed that only 20 μM and 50 μM ANE reduced the activity of NPCs (Fig. 1C). ANE in the concentration range of 2, 5, 10 μM was used for subsequent studies. When NPCs were exposed to 200 μM H_2_O_2_ for 24 h, the activity of nucleus pulposus cells was obviously repressed (Fig. 1D). Interestingly, pretreatment with ANE could effectively attenuate H_2_O_2_-induced inhibition of NPCs activity at a concentration dependent manner (Fig. 1E).

### ANE inhibited oxidative stress and inflammation in H_2_O_2_-induced NPCs

**Fig. 2:**
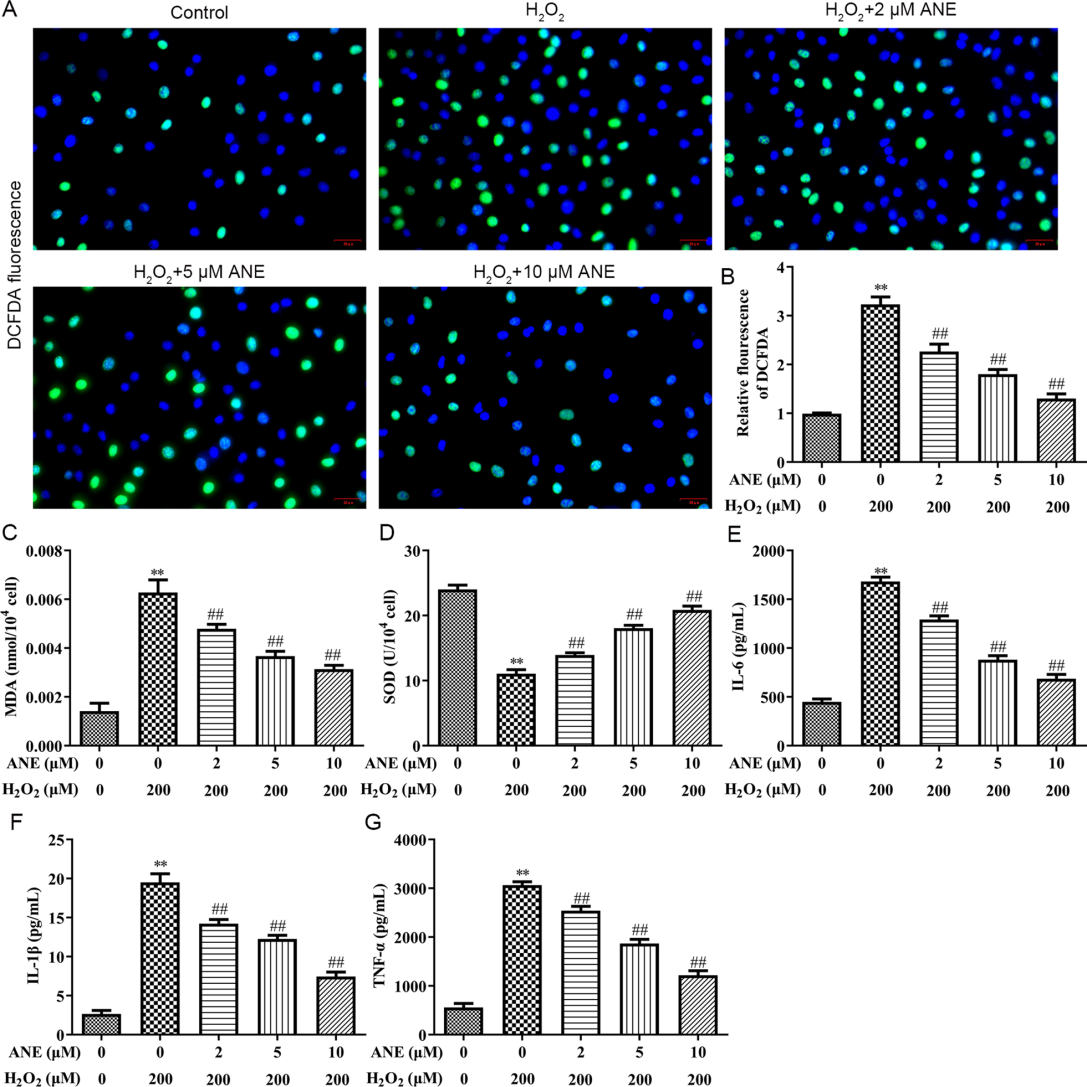
ANE attenuated oxidative stress and inflammation in H_2_O_2_-induced NPCs. **A**, **B** The ROS level was assessed by DCFDA method. **C**–**G** ELISA assay showing MDA, SOD, IL-6, IL-1*β*, TNF-*α* levels. **p < 0.01 versus control group. ##*p* < 0.01 versus H_2_O_2_ treatment group

Next, the effect of ANE on oxidative stress and inflammation was measured in NPCs. NPCs exposed to H_2_O_2_ showed significantly higher DCFDA fluorescence intensity than control group, indicating that H_2_O_2_ obviously raised ROS levels in NPCs (Fig. 2A, B). Moreover, ANE attenuated the upregulation in ROS levels in H_2_O_2_-treated NPCs (Fig. 2A, B). At the same time, MDA and SOD were also detected. The results suggested that MDA level was amplified, while SOD level was lessened in the H_2_O_2_ treated group (Fig. 2C, D). These effects were recovered by ANE pretreatment (Fig. 2C, D). Inflammatory factors is commonly upregulated during IVDD. Furthermore, H_2_O_2_-induced increases of IL-6, IL-1*β* and TNF-*α* levels were attenuated by ANE pretreatment (Fig. 2E–G).

### ANE reduced extracellular matrix degeneration in H_2_O_2_-induced NPCs

**Fig. 3:**
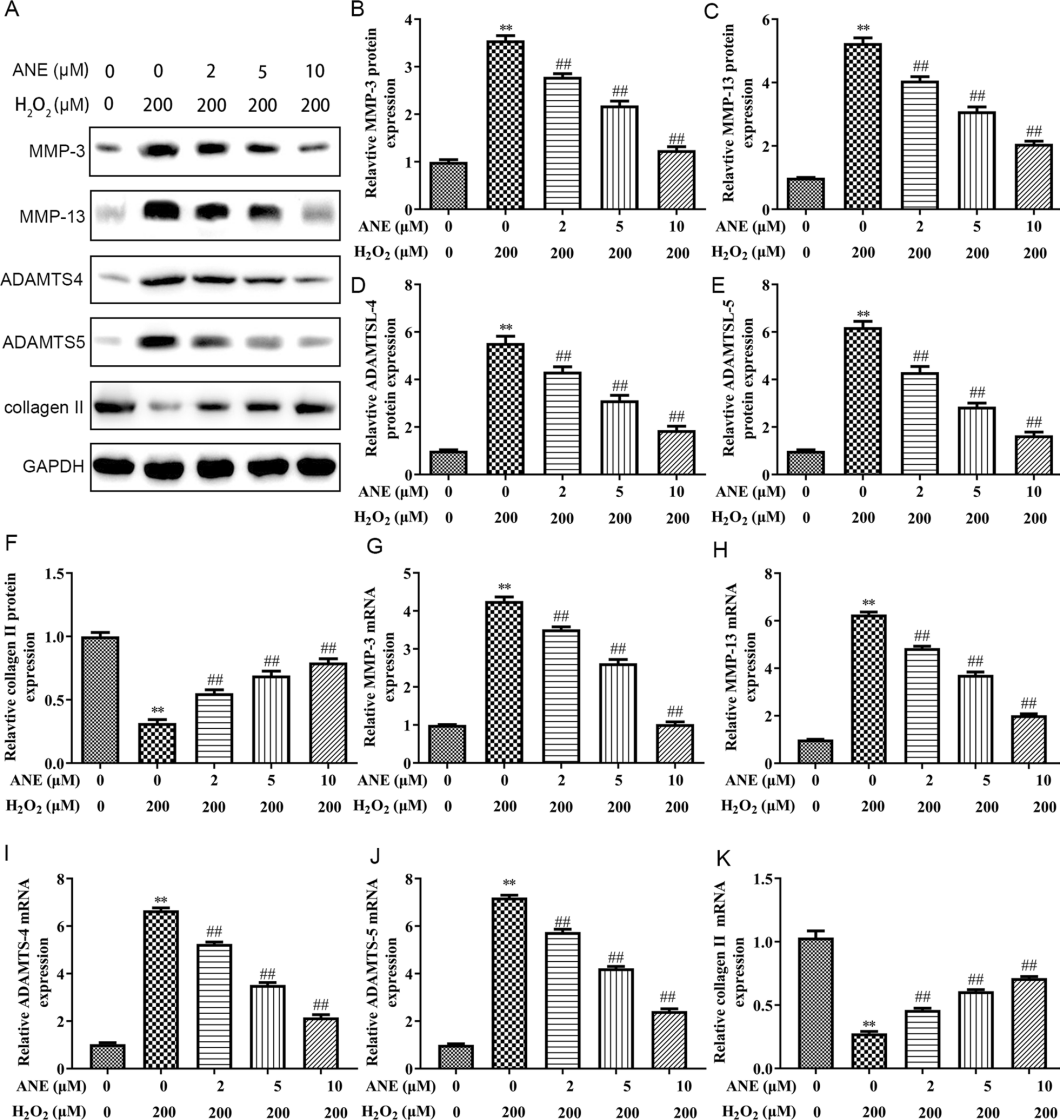
ANE inhibited H_2_O_2_-induced degeneration of extracellular matrix in NPCs. **A**–**F** The protein expression of MMP-3, MMP-13, ADAMTS-4, ADAMTS-5 and collagen II was measured by western blot. **G**–**K** The mRNA expression of MMP-3, MMP-13, ADAMTS-4, ADAMTS-5 and collagen II was measured by RT-PCR. **p < 0.01 versus control group. ##*p* < 0.01 versus H_2_O_2_ treatment group. **p < 0.01 versus control group. ##*p* < 0.01 versus H_2_O_2_ treatment group

The degeneration of extracellular matrix of NPCs is a symbol of IVDD [28]. Therefore, whether ANE could restore the degeneration of extracellular matrix was explored in H_2_O_2_-induced NPCs. MMP-3, MMP-13, ADAMTS-4 and ADAMTS-5 were upregulated in H_2_O_2_-induced NPCs. Nevertheless, ANE blocked the effect of H_2_O_2_ on these enzymes. Conversely, H_2_O_2_ treatment reduced the expression of collagen II, which was repressed by ANE (Fig. 3A–F). The mRNA expression was also assessed. Similarly, mRNA expression of MMP-3, MMP-13, ADAMTS-4 and ADAMTS-5 were significantly increased and collagen II was obviously decreased, and which were reversed by ANE treatment (Fig. 3G–K).

### ANE restrained NOX4/NF-***κ***B signaling pathway in H_2_O_2_-induced NPCs

**Fig. 4:**
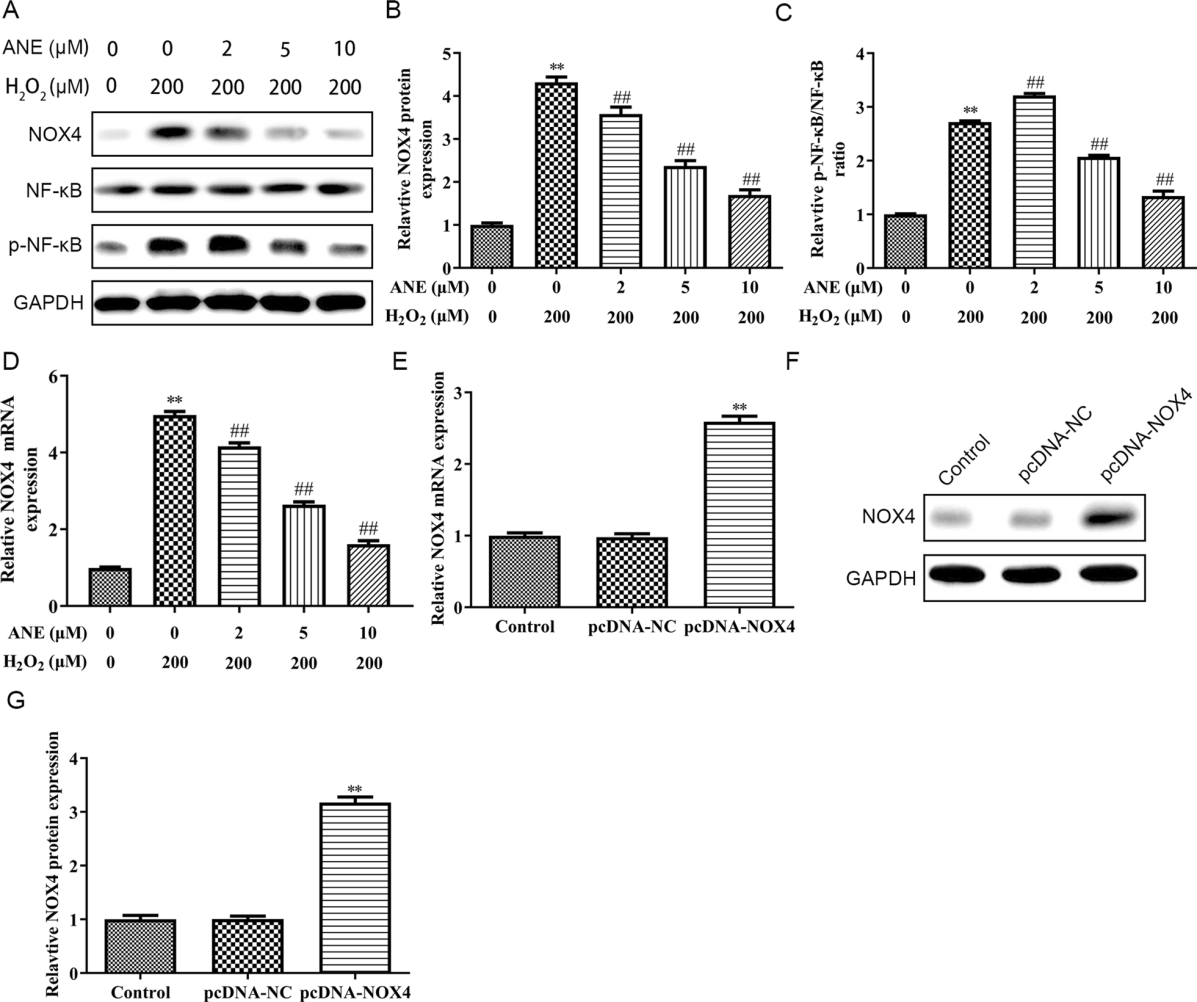
ANE restrained the activation of NOX4/NF-κB signaling pathway. **A**–**C** The protein expression of NOX4, NF-κB and p- NF-κB were assessed by western blot. **D** The mRNA expression of NOX4 was assessed by RT-PCR. **E**–**G** After transfected with or without pcDNA-NC or pcDNA-NOX4, the mRNA and protein expression of NOX4 was detected by RT-PCR and western blot. **p < 0.01 versus control group. ##*p* < 0.01 versus H_2_O_2_ treatment group

Previous studies have demonstrated that NOX4/NF-*κ*B signaling pathway participated in progress of NPCs degeneration, especially through affecting oxidative stress and inflammation [13]. Similarly, in this study, the NOX4 and p-NF-*κ*B/NF-*κ*B ratio were enlarged in H_2_O_2_ exposed NPCs. Interestingly, pretreatment with ANE significantly inhibited NOX4 expression and reduced the p-NF-*κ*B/NF-*κ*B ratio (Fig. 4A–C). Moreover, ANE could recover the enhanced effect of H_2_O_2_ on NOX4 mRNA expression (Fig. 4D). Hence, we suspected that the effect of ANE on NPCs might be achieved by inhibiting the NOX4/NF-*κ*B signaling pathway. To demonstrate our hypothesis, NOX4 was overexpressed in NPCs by transfecting pcDNA-NOX4. Moreover, pcDNA-NOX4 transfection upregulated NOX4 expression in NPCs (Fig. 4E–G).

### ANE antagonized H_2_O_2_-induced degeneration of NPCs by inhibiting NOX4/NF-***κ***B pathway

**Fig. 5:**
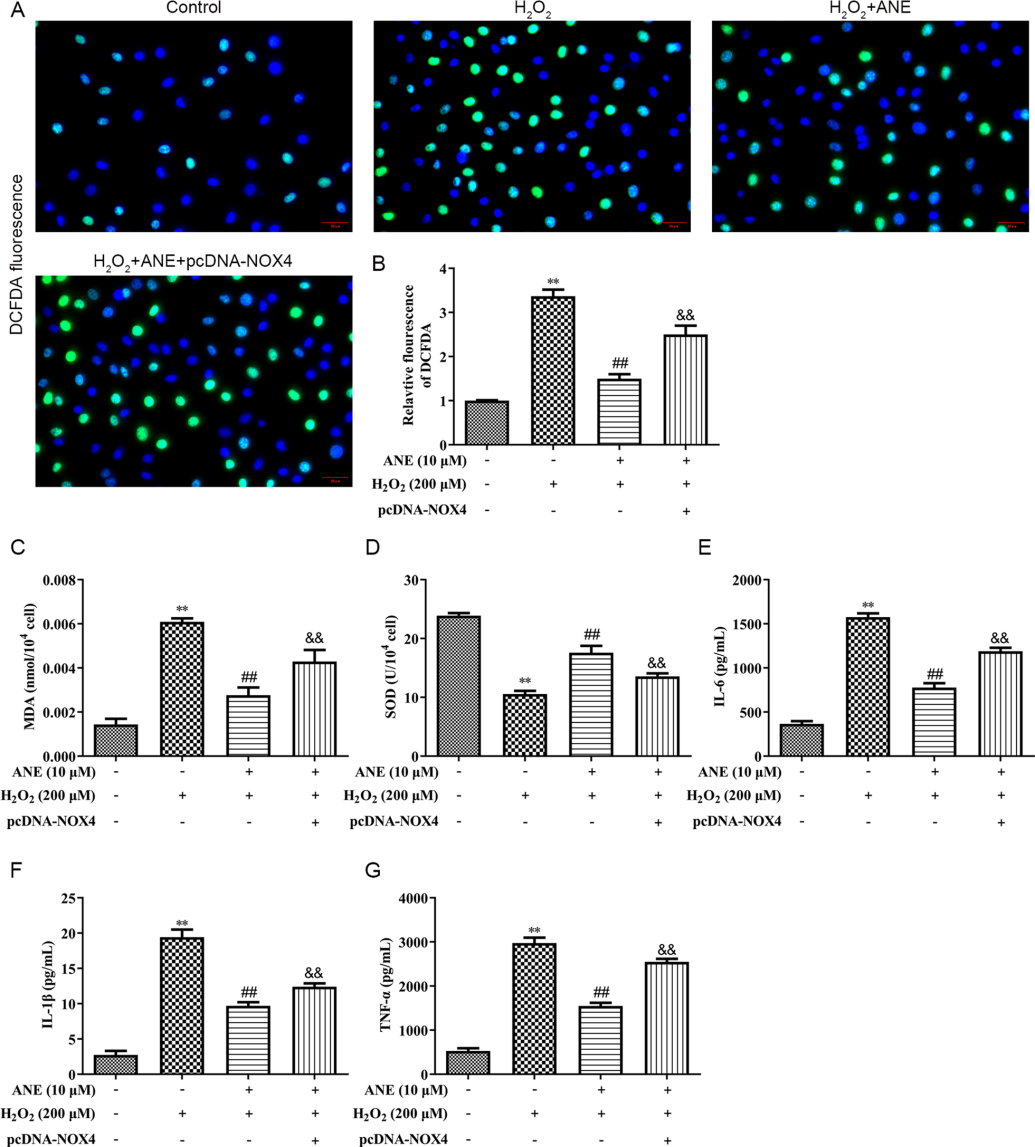
ANE inhibited oxidative stress and inflammation in H_2_O_2_-induced NPCs through inactivating NOX4/NF-κB pathway. **A**, **B** The ROS level was assessed by DCFDA method. **C**–**G** ELISA assay showing MDA, SOD, IL-6, IL-1*β*, TNF-*α* levels. **p < 0.01 versus control group. ##*p* < 0.01 versus H_2_O_2_ treatment group. &&*p* < 0.01 versus ANE + H_2_O_2_ treatment group

**Fig. 6:**
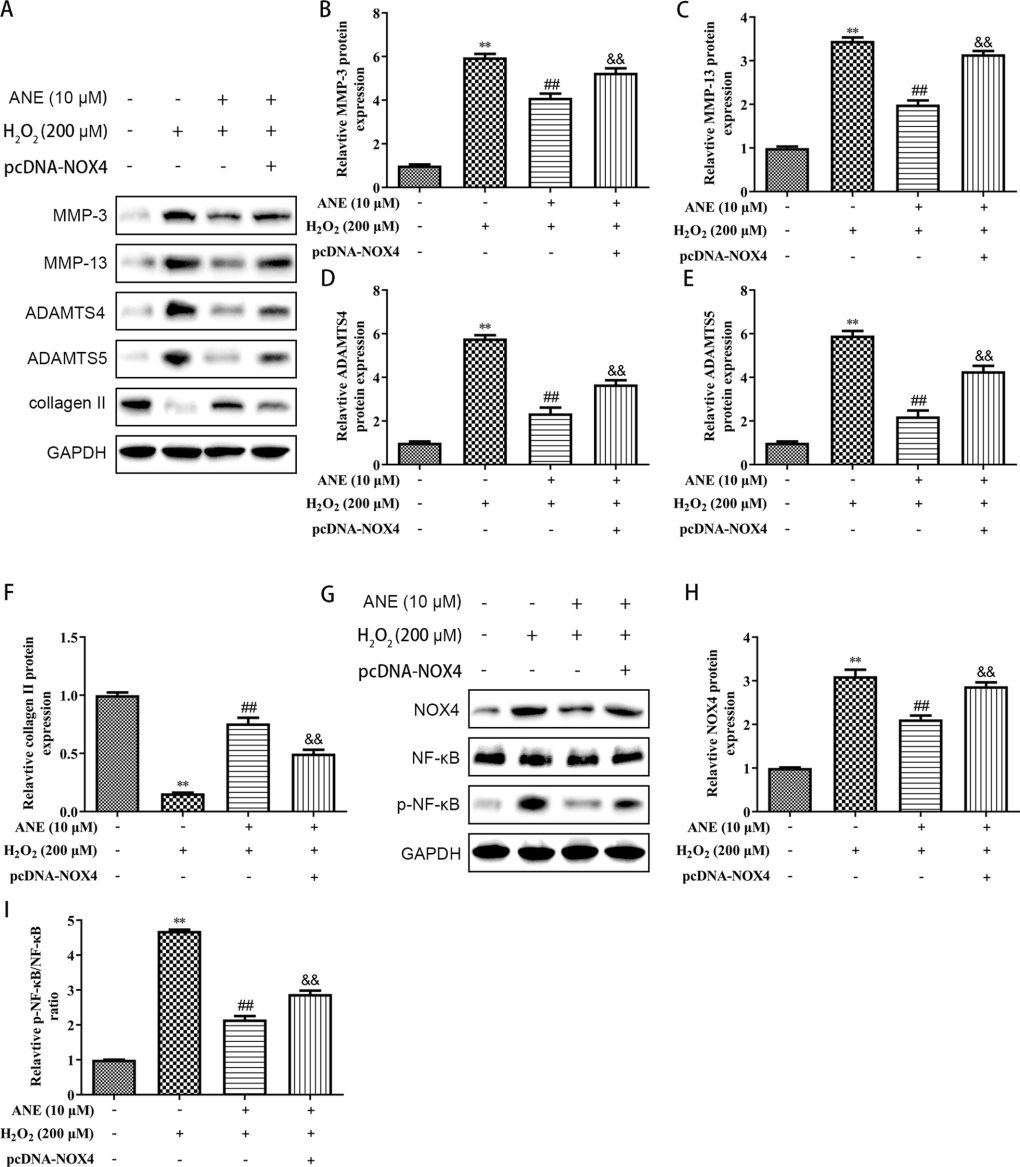
ANE suppressed H_2_O_2_-induced degeneration of extracellular matrix in NPCs through inactivating NOX4/NF-κB pathway. **A**–**F** The expression of MMP-3, MMP-13, ADAMTS-4, ADAMTS-5 and collagen II was measured by western blot. **G**–**I** Western blot showing the expression of NOX4, NF-κB and p- NF-κB. **p < 0.01 versus control group. ##*p* < 0.01 versus H_2_O_2_ treatment group. &&*p* < 0.01 versus ANE + H_2_O_2_ treatment group

It was explored whether ANE protected NPCs by mediating NOX4/NF-*κ*B pathways. Our finding indicated that ANE attenuated ROS and MDA levels and raised SOD level in NPCs induced by H_2_O_2_, which reflected that ANE reduced oxidative stress. Nevertheless, this effect was restored by NOX4 transfection (Fig. 5A–D). ANE repressed the inflammatory factors, which was restored by NOX4 overexpression (Fig. 5D, E). In the H_2_O_2_ treated group, extracellular matrix degeneration was enhanced through the reduction of collagen II and the upregulation of matrix enzymes MMP-3, MMP-13, ADAMTS-4 and ADAMTS-5. Pretreatment with ANE restrained the degeneration of extracellular matrix, while overexpression of NOX4 counteracted the effect of ANE on extracellular matrix (Fig. 6A–F). In addition, ANE-induced the inactivating of the NOX4/NF-*κ*B pathway was also blocked by the overexpression of NOX4 (Fig. 6G–I). Those indicated that ANE resisted oxidative stress, inflammation and extracellular matrix degeneration by restraining NOX4/NF-*κ*B pathway in H_2_O_2_-induced NPCs.

## Discussion

IVDD is the main cause of LBP, but its pathogenesis has not been completely cleared [29]. NPCs play a critical role in IVDD [30]. The recovery activity of damaged NPCs is related to the treatment and prognosis of IVDD [30]. However, oxidative stress promotes the degeneration of NPCs, and then affects the repair of intervertebral disk injury [8, 31]. Hence, it is critical to screen effective and low toxic drugs that could protect NPCs and improve IVDD.

In recent studies, it was showed that ANE possessed a widely biological activities, including anti-inflammatory, antioxidant, anti-bacterial and immune regulation [32–34]. It has been reported that ANE showed neuroprotective effect by providing antioxidant activity and inhibiting apoptosis in MCAO rats [22]. Other studies have reported that ANE could effectively alleviate osteoporosis and osteoarthritis. ANE could reduce the loss of extracellular matrix by inhibiting IL-1*β*/NF-*κ*B pathway during osteoarthritis of mouse [27]. The treatment of ANE could significantly reduce oxidative stress and inflammation in osteoporosis, and further studies showed that ANE improved bone destruction by regulating MAPK-mediated NF-*κ*B signaling pathway [26]. Nevertheless, the therapeutic effect of ANE on IVDD was still unclear. In this study, oxidative stress stimulation of NPCs was achieved by H_2_O_2_ treatment, and then we explored that the damaging effect of H_2_O_2_ on NPCs and whether ANE could protect NPCs under H_2_O_2_. The study revealed that H_2_O_2_ significantly depressed the cell viability of NPCs, and addition of ANE could gradually improve the viability of NPCs under H_2_O_2_. The findings indicated that ANE could decay the damage of H_2_O_2_ to NPCs.

Under normal circumstances, the body has a set of balanced antioxidant system to maintain the balance of free radical metabolism. However, when the body is affected by diseases, exogenous poisons and other factors, ROS is rapidly generated and accumulated, resulting in the imbalance between oxidative stress and antioxidation [35]. Excessive formation of ROS will lead to damage to cell function and pathological changes [36]. MDA is the metabolite of the peroxidation of unsaturated fatty acids in biofilm caused by ROS [37]. SOD is a natural antioxidant enzyme produced by organisms, which removes ROS through disproportionation reaction and blocks the chain reaction of lipid peroxidation [37]. The contents of ROS, MDA and SOD in cells are often used as the representative indicators to assess oxidative stress damage [38]. Melatonin prevented H_2_O_2_-induced decrease of NPCs activity and increase of ROS and MDA levels [39]. Plumbagine could reduce the expression of oxidative stress and inflammatory factors induced by H_2_O_2_ in NPCs [40]. Our study showed that ROS and MDA of NPCs were significantly raised, and SOD was reduced under the effect of H_2_O_2_, indicating the enhancement of oxidative stress. However, ROS and MDA in NPCs pretreated with ANE were significantly repressed, and SOD was amplified, indicating that ANE played a protective role on NPCs induced by H_2_O_2_.

It has been reported that inflammatory mediators could further promote IVDD [14]. IL-6, IL-1*β* and TNF-*α* are important inflammatory factors during IVDD [41]. IL-6 could aggregate inflammatory cells, regulate the overexpression of matrix metallolytic enzymes, promote the degeneration of extracellular matrix and enhance IVDD [42]. It was found that resveratrol improved NPCs growth and degeneration of extracellular matrix by IL-6/JAK/STAT3 pathway [43]. The study has confirmed that IL-1*β* and TNF-*α* are upregulated in IVDD tissues compared with normal intervertebral disk tissues [44]. In this study, H_2_O_2_ stimulated the significant increases inflammatory factors in NPCs. Moreover, ANE restrained IL-6, IL-1*β* and TNF-*α* levels in a concentration dependent manner in H_2_O_2_-treated NPCs. These results indicated that ANE could attenuated inflammation to protect NPCs.

The overexpression of NOX4 was directly related to ROS accumulation, cell aging, apoptosis and the upregulation of metalloproteinases [45]. Silencing the expression of NOX4 has been thought as a new targeting strategy for IVDD [13]. Meanwhile, ROS simulates inflammation and oxidative stress through regulating NF-*κ*B signaling pathway [46]. It was reported that isoquercetin improves oxidative stress and neuronal apoptosis in ischemia–reperfusion model rats and oxygen glucose deprived neurons by inhibiting activation of NOX4/ROS/NF-*κ*B pathway [47]. Feng et al. firstly found the presence of NOX4 in NPCs of intervertebral disk and confirmed that the important roles of NOX4/NF-*κ*B and MAPK in IVDD [13]. In this study, we found that H_2_O_2_ upregulated NOX4 and p-NF-*κ*B in NPCs, which is consistent with the previous study [13]. In this study, after pretreatment with ANE, NOX4 and p-NF-*κ*B were significantly reduced. However, overexpression of NOX4 could counteract the effect of ANE on H_2_O_2_-induced NPCs. These results explained that the protective effect of ANE on NPCs was related with NOX4/NF-*κ*B pathway.

There are still some limitations in this paper. The effect of ANE on the apoptosis of NPCs was not been explored. Moreover, whether ANE could alleviate IVDD has not been explored in vivo. These will be the focus of our future study.

## Conclusion

Our finding for the first time demonstrated that ANE prevented cell viability, reduced oxidative stress injury and repressed inflammatory factors in H_2_O_2_-induced NPCs. Mechanistically, ANE protected NPCs by depressing NOX4/NF-*κ*B signaling pathway. This experimental study is conducive to the clinical application of ANE to alleviate IVDD.
